# Equicarbohydrate partial exchange of kiwifruit for wheaten cereal reduces postprandial glycaemia without decreasing satiety

**DOI:** 10.1017/jns.2016.30

**Published:** 2016-09-14

**Authors:** Suman Mishra, Jinny Willis, Juliet Ansell, John Alexander Monro

**Affiliations:** 1 New Zealand Institute for Plant and Food Research, Palmerston North, New Zealand; 2 Don Beaven Medical Research Centre, Christchurch, New Zealand; 3 Zespri International Limited, Tauranga, New Zealand

**Keywords:** Glycaemic response, Carbohydrate exchanges, Kiwifruit, Appetite, GI, glycaemic index, GR, *Actinidia deliciosa* ‘Hayward’ (marketed as Zespri^®^ Green Kiwifruit), SG, *Actinidia chinensis* ‘Zesy002’ (marketed as Zespri^®^ SunGold Kiwifruit), VAS, visual analogue scale, WB, wheat-based breakfast cereal

## Abstract

Kiwifruit is a carbohydrate food of low glycaemic potency which could potentially be exchanged for starch-based foods in management of postprandial glycaemia. The effect of equicarbohydrate partial exchange of kiwifruit varieties ‘Hayward’ green (GR) and ‘Zesy002’ (SunGold; SG) for a starchy wheat-based breakfast cereal (WB) on the characteristics of the postprandial glycaemic response and satiety was therefore determined. A total of twenty non-diabetic subjects (mean age 36 years; mean BMI 24·5 kg/m^2^) consumed four meals, each containing 40 g available carbohydrate, in random order, after an overnight fast. The meals were: (1) glucose; (2) 70·29 g breakfast cereal; (3) 200 g of GR plus breakfast cereal (30·93 g); and (4) 200 g of SG plus breakfast cereal (27·06 g). Throughout the 180 min postprandial period, capillary blood glucose concentrations were monitored, and satiety rated by a visual analogue scale. Partial kiwifruit substitution of WB significantly reduced postprandial glycaemic response amplitude (glucose, 3·91; WB, 3·66; WB + GR, 2·36; WB + SG, 2·31  mmol/l; least significant difference (LSD) 0·64; *P* < 0·001) and incremental area under the blood glucose response curve (0–120 min) (glucose, 228; WB, 180; WB + GR, 133; WB + SG, 134 mmol/l × min; LSD 22·7; *P* < 0·001). The area between baseline and response remained positive in kiwifruit-substituted meals but became negative after 120 min with glucose and WB, indicating that kiwifruit improved homeostatic control. Kiwifruit substitution of cereal did not significantly reduce satiety. We conclude that either ‘Hayward’ or ‘Zesy002’ kiwifruit may be used in equicarbohydrate partial substitution of starchy staple foods to reduce glycaemic response and improve glucose homeostasis without decreasing satiety.

The acute glycaemic response to digestible carbohydrates in foods has been associated with a range of medical conditions^(^^1^^)^. Blood glucose is known to cause diffuse and progressive damage to tissues and control systems throughout the body in the processes of glycation and glycaemia-induced oxidation^(^^2^^,^^3^^)^. Intense insulin demand evoked by rapid postprandial increases in blood glucose is also believed to be harmful, by placing a burden on the β-cells of the pancreas^(^^4^^)^, and by the action of insulin as an active regulatory hormone. The intense production of insulin can lead to a hypoglycaemic overcompensation after the insulin-stimulated return of blood glucose to baseline. In the hypoglycaemic state, a number of physiological systems may be adversely affected, including appetite control and cognition^(^^5^^)^.

With a growing global incidence of glucose intolerance, associated with obesity and ageing^(^^6^^)^, there is an increasing demand for foods and approaches that can be used in the practical dietary management of postprandial glycaemia. One of the strategies that has been used in dietetic practice is to substitute highly glycaemic carbohydrates in the diet with less glycaemic carbohydrates using the ‘carbohydrate exchange’ system^(^^7^^)^. However, rather than simply substitute one carbohydrate source for another, it would be beneficial if the exchange could be used as an opportunity to improve the nutrient profile of the diet by including nutrient-rich carbohydrate products such as fruit, particularly as many fruit constituents may have a role in ameliorating the effects of glycaemia-induced oxidative stress and inflammation^(^^8^^)^. Even outside the context of carbohydrate exchange, it is valuable for consumers to be able to gauge the likely relative effect that fruit is likely to have on glycaemic response.

In a previous study (JA Monro, H Edwards, S Mishra, D Hedderley and J Podd, unpublished results) of the effects of kiwifruit on the blood glucose response to a glycaemic breakfast cereal, we found that breakfast cereal plus kiwifruit caused a lower glycaemic response than breakfast cereal plus the same amount of sugars, of the same monosaccharide composition, as in the kiwifruit. The kiwifruit was able to significantly reduce the amplitude of the response and the area under the blood glucose response curve between 0 and 120 min, and eliminated the hypoglycaemic reaction between 120 and 180 min. Therefore, the kiwifruit appeared to be having an effect on the blood glucose response profile by mechanisms unrelated to its sugar component, but possibly related to other factors such as organic acids, phenolics and dietary fibre properties that might differ between cultivars.

In diet management to minimise exposure to glycaemia and control energy intake, sugar-rich foods such as fruit should ideally be included by equicarbohydrate substitution, rather than being added to the customary diet. The fruit carbohydrate should replace a carbohydrate of equal or higher glycaemic index to avoid increasing glycaemic response.When the substitution involves kiwifruit partially substituting readily digested starch in a processed cereal-based product, such as wheat biscuit (glycaemic index (GI) = 70), one could expect a substantial lowering of the glycaemic response, because the fruit sugars are approximately half fructose (GI of about 22)^(^^9^^)^, and other components of the fruit further reduce glycaemic response, as discussed above, giving whole kiwifruit a low GI of about 50^(^^9^^)^.

However, a possible consequence of reducing glycaemic impact, and of replacing a proportion of glucose by fructose as a result of partially substituting kiwifruit for breakfast cereal, is that the satiating effect of the meal may be reduced. Appetite is thought to be partially controlled by blood glucose concentrations^(^^10^^)^, and fructose has been reported to be less satiating than glucose^(^^11^^)^. If so, it is possible that kiwifruit substiutution could lead indirectly to reduced satiety and increased energy intakes.

The aim of the present study was, therefore, to determine the extent to which equicarbohydrate partial substitution of a starchy staple by kiwifruit in a meal would lower meal glycaemic impact, and the extent to which satiety would be affected by the substitution. A secondary aim was to compare the relative effectiveness of the kiwifruit cultivars ‘Hayward’ and ‘Zesy002’ with respect to reducing glycaemic impact and maintaining satiety.

## Materials and methods

### Meal components

*Actinidia deliciosa* ‘Hayward’ (marketed as Zespri^®^ Green Kiwifruit) (GR) and *Actinidia chinensis* ‘Zesy002’ (marketed as Zespri^®^ SunGold Kiwifruit) (SG) kiwifruit were provided by Zespri Group Ltd, Tauranga, New Zealand, in a ready-to-eat state of ripeness, and processed within a few days of receipt. They were peeled and the hard apical core removed from the green kiwifruit, then halved and frozen (−20°C). The frozen fruit were allowed to thaw partially and were then crushed to a coarse pulp by briefly (10 s) chopping in a Halde food processor. The pulp was then divided accurately into individual 200 g portions, each stored within a plastic, capped, freezer-proof sundae container until required.

The wheat-based, high-starch, low-sugar breakfast cereal was a commonly consumed commercial whole wheat biscuit (Weet-Bix™, Sanitarium), purchased from a supermarket (WB). Weet-Bix™ are 97 % wholegrain wheat and are made of cooked wheat flakes compressed into a biscuit that disintegrates rapidly on wetting. Weet-Bix™ consists mainly of rapidly digested starch with little sugar (nutrient information: protein 12 % (w/w), fat 1·4 % (w/w), carbohydrate 67 % (w/w) of which sugars make up 2·8 %, dietary fibre 10·5 %), so is a suitable model for a starch-based staple.

The glucose used was dextrose monohydrate (Davis Food Ingredients), which contains 91 % glucose. It is henceforth referred to as glucose, and allowance was made for its water content in all calculations and weight measurements.

### Analyses

#### Available carbohydrates in kiwifruit and meals

The available carbohydrate content of the meals was measured by a validated *in vitro* digestion procedure^(^^12^^)^, using exactly one-tenth of the quantity to be consumed of each of the meals in a volume of 50 ml. The samples were moistened with 10 ml of 1 % NaCl solution and adjusted to pH 2·5 with 1 m-HCl. A volume of 1 ml of 10 % pepsin (Sigma P-7125) solution in 0·05 m-HCl was added and the pot incubated at 37°C for 45 min to simulate gastric digestion. Maleate buffer (5 ml, 0·2 m) was added and the pH adjusted to 6·5 with 0·1 m-NaOH. The volume was accurately made up to the 53-ml mark and pancreatic digestion commenced by adding 1 ml of 5 % pancreatin (Sigma P-7545) solution and 0·1 ml of amyloglucosidase (Megazyme E-AMG). Samples (1 ml) were removed at 0, 20, 40, 80 and 120 min into tubes containing 4 ml absolute ethanol to stop the digestion, and the tubes mixed before storing cold until sugar analysis. The total available carbohydrate content of the digested pulp was measured as reducing sugar at 120 min using a reduced scale modification of the dinitrosalicylic acid method^(^^12^^)^ after an amyloglucosidase–invertase secondary digestion of an aliquot of the ethanolic samples. Sucrose content was measured by the difference in reducing sugars with and without invertase, and the total fructose content was measured by the thiobarbituric acid procedure^(^^13^^)^. Total available carbohydrate measured at each sampling point was plotted to provide a digestion curve for each meal (results not shown). Carbohydrate digested by 120 min was counted as available carbohydrate.

#### Blood glucose

Blood glucose concentrations were measured by finger-prick analysis of capillary blood using HemoCue (Ängelholm) lancets and blood glucose analyser calibrated daily with a glucose reference.

### Formulation of meals

The experiment used four meals, each formulated to contain 40 g available carbohydrate based on the digestive analysis described above, and a water content of 300 ml (Table 1):

(a) Glucose: 40 g of glucose (reference) dissolved in 300 ml water;
(b) WB: breakfast cereal containing 40 g of available carbohydrate (70·29 g WB);
(c) WB + GR: 200 g of ‘Hayward’ green kiwifruit combined with enough breakfast cereal (30·93 g WB) to give a total of 40 g of available carbohydrate;
(d) WB + SG: 200 g of ‘Zesy002’ kiwifruit combined with enough breakfast cereal (27·06 g WB) to give a total of 40 g of available carbohydrate.

Digestive analysis showed that the WB contained 56·9 % available carbohydrate, GR contained 11·2 %, and ‘SG contained 12·3 % available carbohydrate. The amount of WB required to deliver 40 g available carbohydrate was therefore: 40/56·9 × 100 = 70·29 g.

**Table 1.** Weights of kiwifruit and wheaten breakfast cereal (WB) meal components used (g)*

	Meal 1: glucose	Meal 2: WB	Meal 3: WB + GR	Meal 4: WB + SG
Glucose	40	–	–	–
WB	–	70·29	30·93	47·30
GR	–	–	200	–
SG	–	–	–	200
Water (ml)	300	300	120	120

* Kiwifruit were *Actinidia deliciosa* ‘Hayward’ (GR) and *Actinidia chinensis* ‘Zesy002’ (SG).

Based on the carbohydrate contents, the meals (glucose, WB, WB + GR, WB + SG) required for the kiwifruit–WB exchanges to maintain a dietary intake of 40 g available carbohydrate were as shown in Table 1. The distribution of available carbohydrate throughout the meals and meal components is shown in Table 2. The accuracy of the meal formulations was confirmed by *in vitro* digestive analysis of each meal scaled down to one-tenth of the quantity to be ingested (Table 3).

**Table 2.** Available carbohydrate in kiwifruit and wheaten breakfast cereal (WB) meals*

	Meal 1: glucose	Meal 2: WB	Meal 3: WB + GR	Meal 4: WB + SG
Glucose	40	–	–	–
WB	–	40	17·6	15·4
GR	–	–	22·4	–
SG	–	–	–	24·6
Total	40	40	40	40

* Kiwifruit were *Actinidia deliciosa* ‘Hayward’ (GR) and *Actinidia chinensis* ‘Zesy002’ (SG).

**Table 3.** Available carbohydrate digested *in vitro* at 120 min from meal quantities calculated to contain 4·0 g of available carbohydrate based on preliminary *in vitro* analysis, as confirmation of correct meal formulation* (Mean values and inter-duplicate ranges (IDR))

	Glucose	WB	WB + GR	WB + SG
Mean	4·10	4·01	4·02	3·84
IDR	0·19	0·68	0·54	0·18

* The meals were wheaten breakfast cereal (WB), *Actinidia deliciosa* ‘Hayward’ green kiwifruit-substituted WB (WB + GR) and *Actinidia chinensis* ‘Zesy002’ gold kiwifruit-substituted WB (WB + SG).

### Human intervention study

**Fig. 1.**
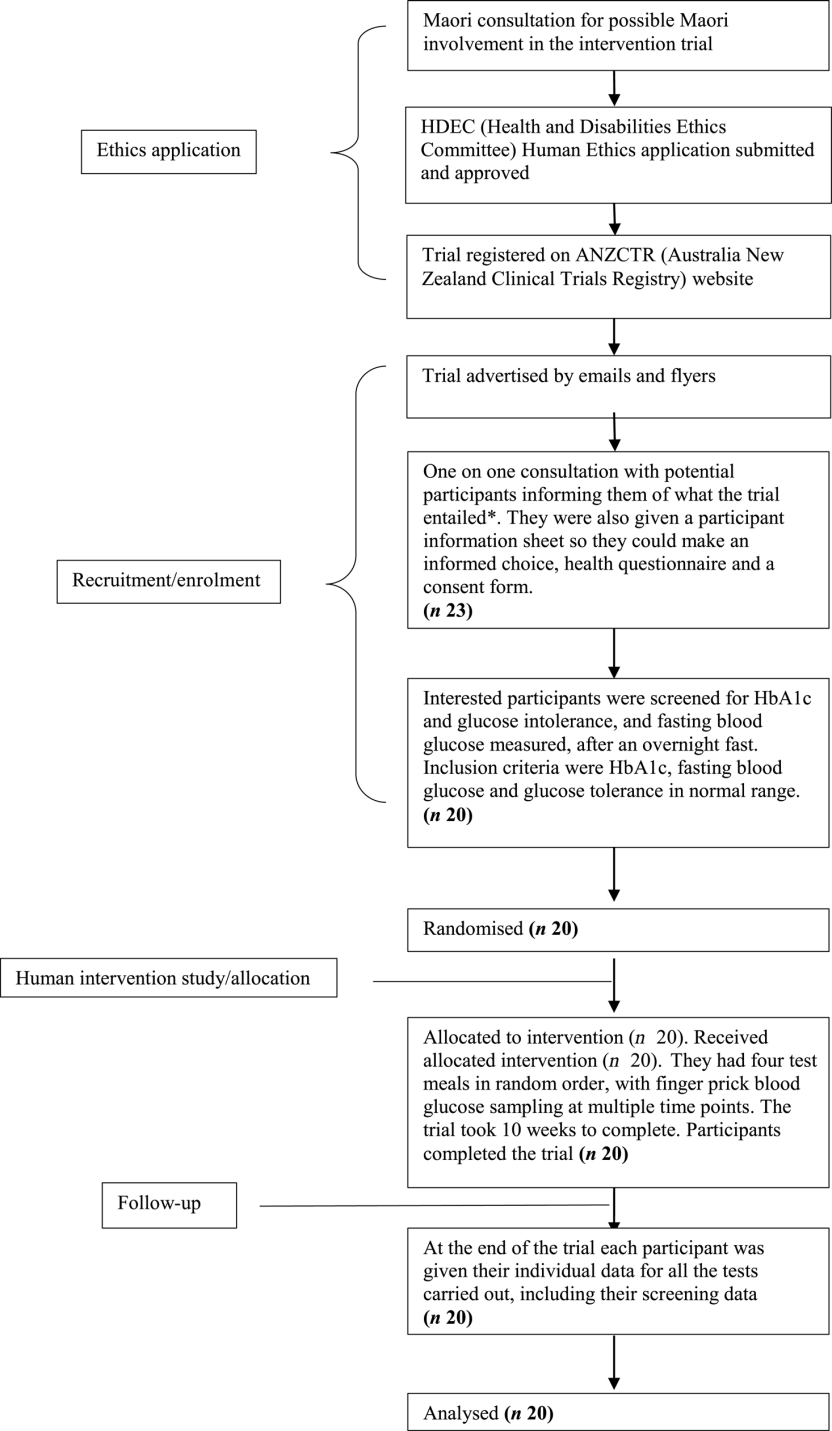
Participant flowchart showing ethical approval, recruitment and intervention processes for a trial of effects of partial substitution of kiwifruit for breakfast cereal on postprandial blood glucose. * The participants were allowed to bring a member of their ‘Whanau’ (support person, family or friend). Family support is very important in Maori culture. HbA1c, glycated Hb.

This study was conducted according to the guidelines laid down in the Declaration of Helsinki and all procedures involving human subjects were approved by the Human and Disabilities Ethics Committee of the New Zealand Ministry of Health (ethics approval number 14/CEN/207). Written informed consent was obtained from all subjects. The trial was registered with the Australia New Zealand Clinical Trials Registry (trial ID: ACTRN12615000222549) (http://www.anzctr.org.au). The participant flowchart shows the ethical approval, recruitment and intervention processes for the trial (Fig. 1). For the CONSORT checklist, see the Supplementary material.

The trial was run as a non-blinded randomised repeated-measures study. It was not possible to blind the subjects to the meals they were consuming. However, the data and statistical analysis was performed by an analyst who was blinded to the treaments. Meal order was randomised for each subject using a computerised random number generator.

#### Subjects

A total of twenty subjects (eight male and twelve female) were recruited by flyer and email. Respondents were interviewed and given an information pack including a description of the study and a consent form. Prospective participants were asked to complete a health questionnaire and provide a capillary blood glucose sample for glucose and glycated Hb analysis. Exclusion criteria included known intolerance of kiwifruit, glucose intolerance as indicated by the fasting blood glucose and glycated Hb (HbA1c), and recent ill health. The characteristics (mean values and standard deviations) of the study group were: age 36·7 (sd 8·1) years, BMI 24·5 (sd 5·2) kg/m^2^, fasting glucose 4·6 (sd 0·4) mmol/l and HbA1c 33·9 (sd 4) mmol/mol.

#### Preparation of meals

The kiwifruit meals were thawed immediately before consuming in a microwave, with care to avoid warming. The meals containing both WB and kiwifruit were well mixed immediately before serving. The meals were consumed with enough water to maintain an approximately equal intake volume in all meals.

#### Glycaemic response

Subjects were asked to consume a moderate meal the evening before sampling, fast overnight from 22.00 hours, avoid exercise on the morning of the test and present themselves at 08.30 hours for the dietary intervention. They were asked to consume the test meals within a 10-min period and avoid physical exertion for 3 h afterward, during which time blood glucose determinations were made. Blood samplings were made immediately before consuming the meals (duplicate, baseline), and at 15, 30, 45, 60, 90, 120 and 180 min after the start of food consumption.

Subjects were instructed simply to eat moderately the night before the experiment so that the influence of a large evening meal would not persist into the testing session. After considering individual differences in body weight, sex and activity of the participants it was concluded that such instructions would be less detrimental to the study, and more simple to implement than trying to impose a dietary regimen, particularly as all subjects were healthy, and normal glucose tolerance had been a condition of inclusion in the study.

#### Satiety

The subjects were asked to rate their appetite at 0, 15, 60, 120 and 180 min, using a four-dimension, 10 cm, visual analogue scale (VAS), with the dimensions: How hungry do you feel? (not at all hungry – extremely hungry); How full do you feel? (not at all full – extremely full); How strong is your desire to eat? (not at all strong – extremely strong); How much food do you think you can eat? (nothing at all – a large amount), based on published research on VAS scales for assessing appetite^(^^14^^)^.

### Statistical analysis

#### Data analyses

Incremental blood glucose responses were calculated by subtracting each individual's baseline fasted blood glucose value from subsequent measurements and then used to determine the incremental area under the blood glucose response curve (iAUC) for each individual. The highest postprandial blood glucose peak for each individual, irrespective of the time of occurrence (nearly all were at either 30 or 40 min), was used to determine the mean peak height for each meal. Data were entered into a Microsoft^®^ Excel spreadsheet for preliminary analysis. For statistical comparison of means (ANOVA), GenStat software was used (version 11.1; VSNi Ltd). Data were analysed using unbalanced ANOVA, testing differences between meals after adjusting for participant and order effects. A power calculation based on the iAUC obtained in a comparision of breakfast cereal and kiwifruit-substituted breakfast showed a sample size of seventeen would be required (*P* = 0·05) in a cross-over design with a power of 80 % to detect a difference.

## Results

### Analysis of kiwifruit

From the digestive analysis of the WB and the kiwifruit, the available carbohydrate contents were determined to be: WB, 56·9 %; ‘Hayward’ green kiwifruit, 11·2 %; ‘Zesy002’ gold kiwifruit, 12·3 %.

The figures were close to values from previous analyses of six cultivars of kiwifruit (Zespri Health Communications; www.zespri.com). The sugars consisted of approximately equal proportions of glucose and fructose, with a lesser sucrose component, in the approximate ratio 2:2:1.

### Digestive analysis of the meals

The available carbohydrate content of WB (56·9 %) was less than given in the nutrient information panel of the commercial product package. In case the disparity was due to insufficient enzyme, or to excess substrate, the WB digestions were repeated with a double concentration of pancreatin and amyloglucosidase, and a smaller quantity of WB (1·5 g rather than 7·0 g). Although the *in vitro* digestion rate increased, the 120 min value of 4·24 g of available carbohydrate was close to the target of 4·0 g.

The unreliability of nutrient information panels as sources of accurate available carbohydrate values for glycaemic response studies has recently been pointed out^(^^15^^)^. Much of the discrepancy between measured carbohydrate values and nutrient panel values may result from differences in analytical approaches to the measurement of ‘available’ carbohydrate. Analysis of starch for inclusion in ‘carbohydrate’ values, assumed to represent available carbohydrate, often involves conditions such as heat treatment^(^^16^^)^, which are not part of the human digestive process. Also, extended digestion times of 16 h are used in the measurement of available carbohydrate, whereas the transit time through the small intestine is about 4 h, with most of the carbohydrate digestion in the proximal half. The method used in the present research has been validated with respect to glycaemic response, and used digestion conditions and a duration that approximated that in the human gut^(^^17^^)^.

After the meals had been formulated based on the above available carbohydrate analyses, they were checked by further digestive analysis to confirm that they would deliver a 40 g dose of available carbohydrate. Scaled down to a tenth of the quantity ingested, all meals released a similar amount of available carbohydrate during *in vitro* digestion, corresponding to an available carbohydrate intake of 40 g (Table 3). The meals were therefore equicarbohydrate, as intended.

### Blood glucose responses

All twenty subjects completed the trial and the results from all of them were used in the data analysis.

The between-subject variations in blood glucose responses were large, as is typical of such studies, but no outliers were removed.

**Fig. 2.**
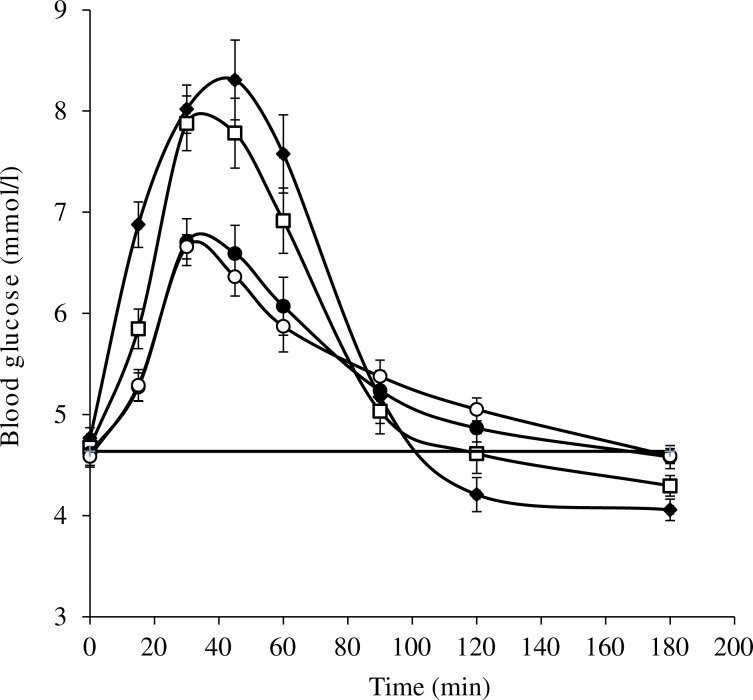
Blood glucose responses induced by equal available carbohydrate meals: glucose (40 g; ♦); wheaten breakfast cereal (WB; □); WB + *Actinidia deliciosa* ‘Hayward’ green kiwifruit (●); WB + *Actinidia chinensis* ‘Zesy002’ gold kiwifruit (○). –––, Baseline. Values are means, with standard errors represented by vertical bars.

The different meals induced blood glucose responses that were clearly distinctive (Fig. 2).

### Amplitude

The 40-g glucose reference caused the highest amplitude postprandial response, followed by a hypoglycaemic overshoot starting at 120 min (Table 4). The WB produced a response of similar amplitude to the reference. Partial substitution of WB by both the kiwifruit cultivars caused a statistically significant, substantial and similar reduction in the response amplitude of about 40 % compared with that for the WB alone.

**Table 4.** Peak incremental blood glucose concentrations (mmol/l) in response to consuming 40 g glucose (reference) and wheaten breakfast cereal (WB), *Actinidia deliciosa* ‘Hayward’ green kiwifruit (GR) plus WB (WB + GR), and *Actinidia chinensis* ‘Zesy002’ gold kiwifruit (SG) plus WB (WB + SG) (Mean values with their standard errors)

	Meals					
	Glucose	WB	WB + GR	WB + SG	LSD	*P*
Mean	3·91	3·66	2·36	2·31	0·642	<0·001
sem	0·26	0·28	0·21	0·13		

LSD, least significant difference.

### Areas between baseline and curves

Areas between the baseline and the blood glucose response curves include both hyperglycaemic and hypoglycaemic periods during the 180-min period after food consumption (Table 5). Over the whole 180-min testing period there were differences between the mean net areas, with the area for the GR-substituted WB being 14 % less than the area for WB, and the area for the SG-substituted WB being about 9 % less than the WB area (Table 5). However, the difference between the glucose reference and GR-substituted WB was 25 % and the difference between the glucose reference and SG-substituted WB was 20 % for the 0–180 min period. Between 0 and 120 min, the incremental areas between the responses to all meals were positive, but significantly different (*P* < 0·001). Between 120 and 180 min, the glucose reference and WB were negative (hypoglycaemic), while the blood glucose responses to WB partially substituted with either GR or SG kiwifruit were slightly positive, and the differences were significant (*P* < 0·005; Table 5).

**Table 5.** Area (mmol/l × min) between blood glucose response curve and baseline during different periods after consuming meals containing glucose, wheaten breakfast cereal (WB), *Actinidia deliciosa* ‘Hayward’ green kiwifruit-substituted WB (WB + GR) and *Actinidia chinensis* ‘Zesy002’ gold kiwifruit-substituted WB (WB + SG) (Mean values with their standard errors)

	Meals					
	Glucose	WB	WB + GR	WB + SG	*P*	LSD
0–180 min						
Mean	188·0	164·9	140·3	149·1	0·012	26·8
sem	15·7	19·6	15·0	12·8		
% Difference from glucose	0·0	−12·3	−25·4	−20·7		
% Difference from WB	14·0	0·0	−14·9	−9·6		
0–120 min						
Mean	227·7	179·7	133·3	134·3	<0·001	22·7
sem	18·21	18·74	13·85	10·39		
% Difference from glucose	0·0	−21·1	−41·5	−41·0		
% Difference from WB	26·7	0·0	−25·8	−25·3		
120–180 min						
Mean	−39·7	−14·8	7·04	14·9	<0·001	11·5
sem	6·40	7·09	6·37	5·91		
% Difference from glucose	0	62·7	117·7	137·5		
% Difference from WB	−167·8	0	147·5	200·5		

LSD, least significant difference.

### Measures of satiety and appetite

**Fig. 3.**
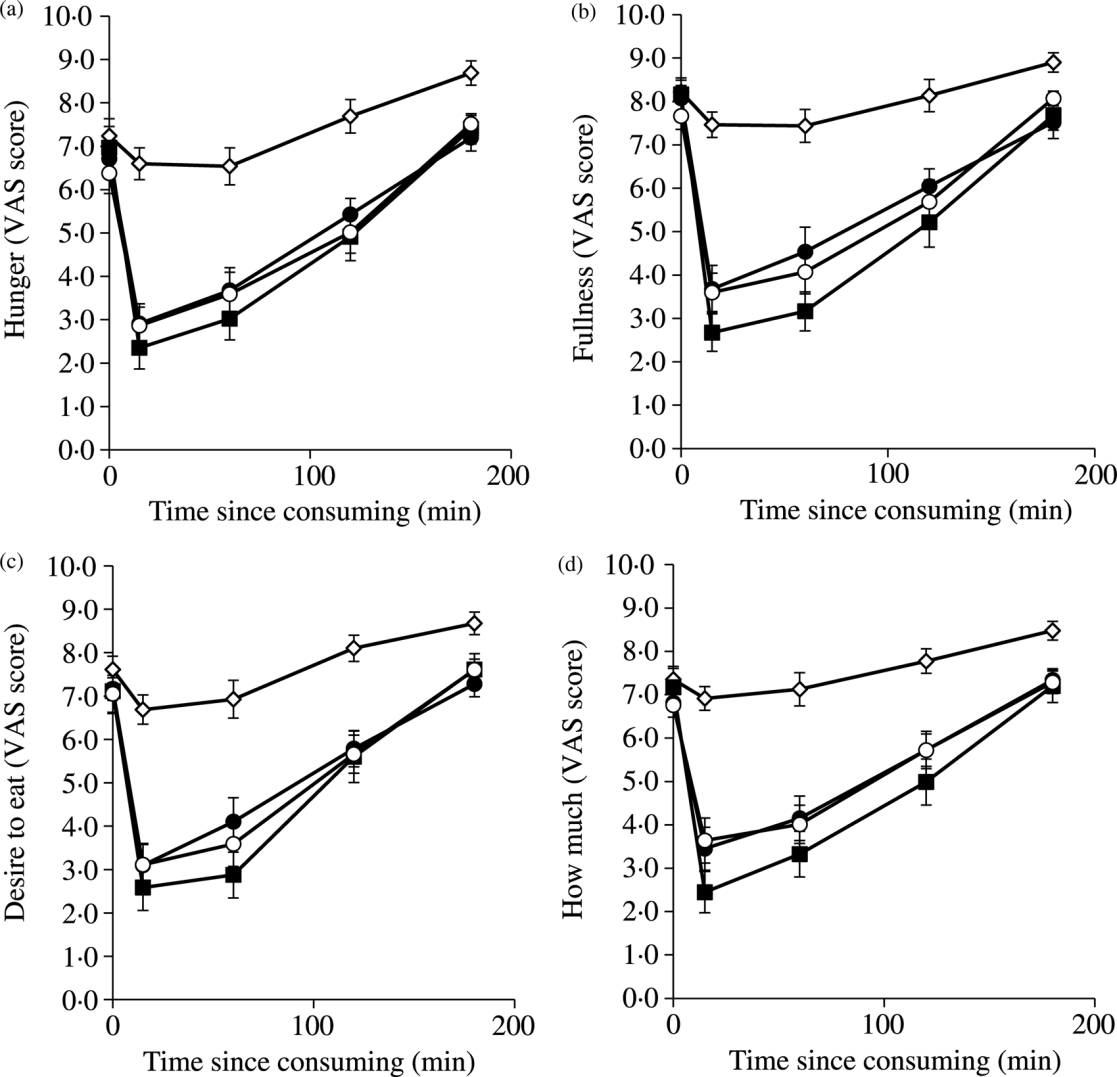
Visual analogue scale (VAS) scores out of 10 on four dimensions of appetite in response to the questions: (a) ‘How hungry do you feel?’; (b) ‘How full do you feel?’ (score subtracted from 10); (c) ‘How strong is your desire to eat?’; (d) ‘How much food do you think you could eat?’ after consuming meals consisting of: glucose (◊); wheaten breakfast cereal (WB; ■); WB + *Actinidia deliciosa* ‘Hayward’ green kiwifruit (●); WB + *Actinidia chinensis* ‘Zesy002’ gold kiwifruit (○) (least significant differences 15 min, 1·11, *P* < 0·001; 60 min, 1·22, *P* > 0·001; 120 min, 0·94, *P* > 0 0·001; 180 min, 0·67, *P* < 0·001). Values are means, with standard errors represented by vertical bars.

All four subjective VAS measures of satiety and appetite gave very similar profiles (Fig. 3). The scores on the four dimensions were also combined for each meal, for each individual, to obtain individual overall satiety scores that were used for statistical analysis. The VAS scores are directly related to appetite/hunger: the higher the score, the greater the hunger. The glucose reference had a small impact. In the meals in which kiwifruit substituted part of the WB, and in the WB meal, all of which contained 40 g of available carbohydrate, appetite immediately after consuming the foods was suppressed to a similar degree, but slightly though not significantly more by WB than by kiwifruit-substituted WB. Given that over 50 % of the WB had been substituted by kiwifruit, and that the volumes of the meal had been made similar by adjusting water intake, satiety was reduced little by partially exchanging cereal for kiwifruit.

The appetite ratings tracked over time showed that the kiwifruit-substituted WB meals were able to sustain satiety as well as the WB alone at all measurement points after food consumption, and at all these points were significantly better than the equal carbohydrate glucose dose (Fig. 3).

## Discussion

The present study was designed to determine the effects of including kiwifruit in a breakfast cereal meal by equicarbohydrate partial substitution of kiwifruit for the breakfast cereal. The results show that kiwifruit-based carbohydrate exchanges led to a substantial reduction in glycaemic response. This reduction is likely to have resulted from a combination of the effects of digestion-resistant non-carbohydrate components of kiwifruit with the effect of replacing starch with fructose, which has a much lower intrinsic glycaemic potency than glucose derived from starch. In the present study the meals were made equicarbohydrate with respect to carbohydrate released after digestion, so the increase in carbohydrate mass on hydrolysis of starch to sugar had been allowed for.

In a previous study we showed *in vitro* that under gastrointestinal conditions, and at relevant concentrations, cell wall remnants (dietary fibre) of kiwifruit were able to reduce glucose diffusion rate and luminal mixing, each by about 40 %^(^^18^^)^. Both processes are involved in the transfer of digestion products to the gut wall for absorption. At a micro level, reduced diffusion and mixing will retard processes such as enzyme movement and end product transfer, resulting in a reduced rate of digestion. In addition to dietary fibre a range of secondary metabolite compounds not measured in the present study have been shown to be present in kiwifruit^(^^19^^)^, and some of them may affect glycaemic response. Organic acids in kiwifruit flesh^(^^20^^)^, which has a pH of about 3·4, may contribute to a reduced glycaemic response by lowering rates of gastric emptying^(^^21^^)^. Phenolics present in kiwifruit may also contribute to a suppression of the glycaemic response by a number of mechanisms, as has been demonstrated in a range of plant-based foods^(^^22^^)^. Such properties are not unique to kiwifruit, but are likely to be manifested by many of the fruits and vegetables that contribute functionally beneficial components to the diet.

Nonetheless, kiwifruit have a very high density of nutrients such as vitamin C^(^^23^^)^, and as they become widely consumed in Asian countries, which are also facing an epidemic of diabetes^(^^24^^–^^26^^)^, it is important to show how they may be incorporated into the diet to increase nutrient intake while not exacerbating glycaemia. Equicarbohydrate exchange, as the present paper shows, is an effective strategy to concurrently achieve both an increase in nutrient intake and a decrease in glycaemic impact. It may therefore be beneficial in both decreasing exposure to glycaemia and in protecting against the long-term damage that it may cause.

It has been suggested that fructose is highly lipogenic, partly because fructose does not have the satiating capacity of glucose^(^^11^^)^. However, the results presented here have shown either that the quantity of fructose consumed in two kiwifruit was not sufficient to reduce satiety when exchanged for glucose, or that other kiwifruit components with satiating activity counteracted any reduction in satiety.

There is also evidence that fructose is highly lipogenic because it takes a more direct metabolic pathway to lipogenesis than glucose. It has even been suggested that high fructose intakes are partly responsible for the current epidemic of obesity and the metabolic syndrome^(^^11^^)^. In the context of kiwifruit-based carbohydrate exchanges, the dietary increase in fructose as a result of including two kiwifruit per d would be about 12 g/d. As long as other sources of fructose in the diet are not excessive, an additional 12 g is unlikely to be harmful, particularly when, as in the present case, it is the result of almost isoenergetic equicarbohydrate exchange of two carbohydrate foods, with no loss of satiety. Furthermore, there is evidence that fructose acts cooperatively to encourage glucose disposal, and that there is nothing intrinsically harmful about fructose *per se* when consumed in the quantities and in the glucose:fructose ratios that would be supplied by fruit and vegetables in a healthy diet^(^^27^^)^.

In addition to lowering dietary glycaemic impact, inclusion of kiwifruit by partial substitution of a starch-based staple such as noodles or rice would alter the nutrient profile of the diet, increasing intakes of vitamin C, K, vitamin E and antioxidants and a range of other nutrients, all in a low-energy-density format of fresh fruit.

As all meals used in the present study were of equal available carbohydrate content (40 g), it is possible to estimate GI values for the carbohydrates in the meals from the 120 min iAUC given in Table 5. Compared with glucose (GI = 100) the GI of the wheat biscuit was 78·9, and after partial substitution with kiwifruit the GI values were reduced to 58·5 for WB + GR and 59 for WB + SG. The GI values are estimates because the carbohydrate quantity used was 40 g, whereas the standard quantity for determination of GI is stipulated as 25 g, or more commonly 50 g of available carbohydrate (Australian Standard^®^ AS 4694-2007)^(^^28^^)^. However, the present study was about the relative glycaemic effects of meals expressed as glucose equivalents, because people eat foods and meals, and not simply the carbohydrates in them. The results will be most accurate and relevant when based on customarily consumed intakes.

The serving size given on the wheat biscuit nutrient information panel is 33 g (two biscuits). With an available carbohydrate content of 56·9 % measured in the present study, a serving would deliver 18·8 g of available carbohydrate. A 50 g intake of available carbohydrate would require that 2·7 servings be consumed, compared with 2·1 servings for the 40 g available carbohydrate intake used in the present study. Also, GI as a measure of relative glycaemic potency of carbohydrate in a food is likely to be slightly more accurate with a glucose reference intake of 40 g than 50 g, because error due to the non-linearity of the glucose equivalent dose–glycaemic response curve will be less at lower glucose equivalent doses^(^^29^^)^.

Overall, the results indicate that inclusion of kiwifruit in diets by equicarbohydrate substitution of highly digestible starch components, to enrich the diets functionally and nutritionally, will also lead to glycaemic benefits. At the least, despite the perception of kiwifruit as a sweet-flavoured fruit, there is no indication that consuming it will have a negative glycaemic impact when it is introduced to a diet without increasing the available carbohydrate load.

This paper has been about the interaction of kiwifruit and breakfast cereal in glycaemic response. Although it is relevant to dietary management in diabetes, the gut-level mechanisms involved are likely to be very similar in both diabetic and non-diabetic individuals. Thus the relative effects of kiwifruit are likely to be similar, although the absolute blood glucose responses would no doubt be greater in a diabetic cohort. The promising findings in the research with healthy individuals presented here should, nonetheless, be repeated with a diabetic cohort.

A possible limitation of the present study is that the kiwifruit was stored as a frozen slurry, to standardise serving size and stage of ripeness, whereas kiwifruit are mostly consumed as whole fruit. However, it is most likely that the kiwifruit consumed as fresh fruit would have had a lower glycaemic effect than observed for the frozen kiwifruit preparations consumed in the present study, due to the influence of fruit tissue structure, which suggests that the present findings are robust. In further studies concurrent measurement of insulin responses may help better interpretation of the effects of kiwifruit, and there remains a need for research to quantify the roles of the various non-available carbohydrate components – phenolics, dietary fibre, organic acids – whose glycaemic response-lowering effects appear to be superimposed on the effect of substituting fructose for glucose derived from starch.

## Supplementary material

For supplementary material accompanying this paper visit http://dx.doi.org/10.1017/jns.2016.30 click here to view supplementary material
